# Surface-driven endocrine activity of nanoplastics: polymer- and size-dependent estrogen and androgen receptors modulation without steroidogenesis perturbation

**DOI:** 10.1186/s12989-026-00692-7

**Published:** 2026-06-10

**Authors:** Nikolina Peranić, Lucija Božičević, Nikolina Kalčec, Korinna Altmann, Jana Hildebrandt, Carlos Cuestas Ayllon, Raquel Portela, Jesús M. de la Fuente, Miguel A. Bañares, Ivana Vinković Vrček

**Affiliations:** 1 Institute for Medical Research and Occupational Health, Ksaverska cesta 2, Zagreb, 10000 Croatia; 2 Bundesanstalt für Materialforschung und prüfung, Unter den Eichen 87, 12205 Berlin, Germany; 3 Instituto de Nanociencia y Materiales de Aragón, CSIC-Universidad de Zaragoza and CIBER-BBN, c/Pedro Cerbuna s/n, Zaragoza, 50009 Spain; 4 Institute for Catalysis and Petrochemistry, CSIC, C. de Marie Curie 2, Cantoblanco, E 28049 Madrid, Spain; 5 Faculty of Medicine, University of Rijeka, Braće Branchetta 20, Rijeka, Croatia

## Abstract

Growing production and use of plastics have led to significant environmental pollution including the formation and accumulation of plastic nanoparticles (PNPs). Due to their small size, PNPs easily enter the human food chain; however, humans are also exposed to plastics through other consumer pathways, such as the use of cosmetic products. Despite considerable efforts to investigate the potential adverse effects of plastics, their impact on human health is not yet fully understood. In particular, endocrine disruption has emerged as a potential mechanism underlying reported reproductive and hormonal effects of micro- and nanoplastics. We applied an OECD-aligned in vitro test guidelines (TGs) to a factorial panel of eight PNPs spanning four common polymers (polystyrene (PS), polyethylene (PE), polypropylene (PP), polyethylene terephthalate (PET)) with size-resolved materials and polymer-matched mixtures. Thus, estrogen receptor a (ERα) transactivation (TG 455), androgen receptor (AR) transactivation (TG 458, antagonist mode), and H295R steroidogenesis (TG 456) assays were performed using HeLa-9903, AR-EcoScreen GR KO M1, and NCI-H295R cell models, respectively. Across 0.1–10 mg L⁻¹, no cytotoxicity was observed. PENPs (350 nm) and PPNPs (180 nm) acted as ER agonists, whereas PPNPs (50 and 180 nm) and PENPs (350 nm) antagonized AR; PSNPs and PETNPs showed no activity when tested individually. Notably, several mixtures elicited ER and AR responses even when constituent singles were inactive, indicating mixture-dependent potentiation. In contrast, the H295R assay did not meet the OECD decision rule for altered steroidogenesis: sporadic shifts in pathway intermediates did not propagate to estradiol or testosterone. Altogether, the data support a surface- and polymer-dependent, receptor-proximal mode of action for PNPs and highlight mixture effects as a critical, underappreciated driver. These results move endocrine hazard evaluation beyond polystyrene surrogates and provide decision-useful guidance on which polymers/sizes and mixture contexts merit priority in exposure monitoring and risk assessment.

**Supplementary Information:**

The online version contains supplementary material available at 10.1186/s12989-026-00692-7.

## Introduction

Production of plastics - multifunctional, resistant, easy-to-process, and affordable material - has been consistently growing and is projected to double over the next 20 years in a business-as-usual scenario [1, 2]. However, their use has become tainted by plastic pollution, which is considered one of the most concerning environmental problems due to its abundance and persistence in the aquatic environment [3]. Plastics in the environment undergo slow photo-, chemical, physical and biological degradation and fragmentation to micro- and nanoparticles [4, 5]. Indeed, the increasing environmental pollution with plastic nanoparticles (PNPs) has amplified global concerns. PNPs can be released into the environment as “primary” or intentionally manufactured particles (e.g. paints, adhesives, electronics and cosmetics) and/or emerge as “secondary particles” because of the degradation of larger plastic objects [6–8]. Chronic exposure to plastic is already an issue of great concern for our health - from nanoplastics in cosmetics and synthetic fibers in clothing, to plastic particles in water and in the air [9, 10]. Although numerous studies have shown that the human body interacts with plastics, the consequences and effects on human health remain unclear. Human health risk assessment for PNPs pollution is currently very challenging due to lack of reliable and sufficient data on both toxicological hazard and human exposure [11]. The behavior of these particles depends on their size, shape, polymer type, surface functionalization, and the type of molecules they interact with [12]. Size is considered a key factor influencing the absorption, bioaccumulation, biodistribution, and thus the toxicity of PNPs. Smaller particles have a larger specific surface area, are more reactive, and can more easily cross biological barriers, which suggests they may pose a greater risk than plastic microparticles (PMPs) [13]. Despite this, the number of studies investigating PNPs is far lower than those focusing on PMPs, which can be attributed to the lack of adequate techniques for visualizing and quantifying PNPs in complex biological matrices. Furthermore, although toxicity has been shown to depend on the particle shape, most studies use spherical polystyrene PMPs and PNPs due to their availability and ease of surface functionalization [14–16]. Existing literature on the effects of PMPs and PNPs has mainly addressed cytotoxicity, immunotoxicity, and genotoxicity, while only a limited number of studies report their effects on reproductive health [17]. The endocrine system regulates reproduction, development, and metabolism, and is highly sensitive to exogenous perturbations. Experimental studies have demonstrated that exposure to PMPs and PNPs can alter endocrine-related outcomes, including reduced testosterone levels, impaired spermatogenesis, and decreased fertility in rodent models exposed to polystyrene particles [18, 19]. Additionally, negative effects on oocyte maturation, fertility, and embryo development have been confirmed in female mice, along with uterine fibrosis caused by oxidative stress [20, 21]. These findings suggest that endocrine-mediated mechanisms may contribute to observed reproductive toxicity. In addition, nanomaterials are known to interact with biological systems through surface-driven processes, including adsorption of biomolecules and formation of protein coronas, which can modulate receptor signaling pathways and ligand availability [12]. Such interactions may influence nuclear receptor activity, including estrogen and androgen receptors, even in the absence of direct chemical toxicity. Therefore, assessing endocrine activity using receptor-based assays and steroidogenesis models provides a mechanistically relevant and regulatory-aligned framework for evaluating potential hazards associated with nanoplastics.

Even though there is growing interest in how PNPs affect biological systems, most studies to date have focused on the effects of individual plastic particles while research on combined effects of PNPs mixtures, remains scarce. In other studies on complex mixtures, it has been shown that PNPs with chemicals or other nanomaterials can exert enhanced additive or synergistic effects on cellular toxicity, proliferation, and inflammatory responses, whereas individual components often show little to no effect alone [22–24]. This highlights the importance of assessing combined impacts rather than single substances alone. We previously reported estrogenic activity of differently sized PNPs composed of multiple polymers, but could not disentangle the relative contributions of size versus polymer chemistry to biological responses [25]. To address this gap, the present study applies a comparative, factorial design encompassing four environmentally prevalent polymers - polystyrene (PS), polyethylene (PE), polypropylene (PP), and polyethylene terephthalate (PET) [26, 27] - together with size-resolved PSNPs (50, 150 and 350 nm), PENPs (50 and 350 nm), PPNPs (50 and 180 nm) and PETNPs (80 nm) to explicitly evaluate size effects [28]. The selected PNPs sizes were constrained by the availability of well-characterized reference materials for non-PS polymers. While PSNPs were available in a size-resolved series (50, 150, 350 nm), equivalent size-matched materials for PP and PET are not commercially or experimentally standardized. Therefore, PPNPs with size of 180 nm and PETNPs with size of 80 nm were included as the closest available representatives within the nanoscale range, enabling inclusion of these environmentally relevant polymers while maintaining material quality and reproducibility. This reflects a broader limitation in nanoplastics research, where non-PS reference materials remain scarce.

We evaluate endocrine-disrupting potential of PNPs and their mixtures (consisted of equal concentrations of each PNP component) across three complementary in vitro models aligned with OECD guidance for endocrine screening, capturing estrogen receptor (ER)-mediated activity, androgen receptor (AR)-mediated activity, and effects on steroidogenesis using HeLa-9903, AR-EcoScreen GR KO M1, and NCI-H295R cell models, respectively [29–31]. By systematically separating the effects of polymer type and particle size within standardized assay frameworks, this work (i) clarifies structure–activity relationships governing receptor-mediated responses to PNPs, (ii) benchmarks endocrine outcomes across polymers commonly encountered in the environment, and (iii) provides decision-useful evidence to prioritize polymers and size ranges for exposure monitoring and risk assessment. The resulting data aim to move the field beyond PS surrogates toward polymer- and size-aware hazard evaluation.

## Materials and methods

### Characterization of PNPs

All plastic nanoparticles were supplied as suspensions and used without further modification. The 50 nm-sized PENPs (PE50), PSNPs in size of 50 nm (PS50), 150 nm (PS150) and 350 nm (PS350), and 50 nm-sized PPNPs (PP50) were purchased from CD Bioparticles (Shirley, NY, USA) at stock concentration of 10,000 mg/L and were suspended in 0.1% Tween 20 in distilled water containing 2 mM NaN₃. PENPs in size of 350 nm and 80 mg/L stock concentration (PE350) and PPNPs in size of 180 nm and 40 mg/L stock concentration (PP180) were suspended in water and produced at the Bundesanstalt für Materialforschung und -prüfung (BAM, Berlin, Germany) according to previously published procedure [32]. The 80 nm-sized PETNPs (PET80) at 1,600 mg/L stock concentration were suspended in water and prepared at Instituto de Catálisis y Petroleoquímica (Madrid, Spain) within PlasticsFatE project following a published procedure [33, 34]. All NPs were plain and used without further modification. Information on size and surface potential of PNPs and their mixtures was obtained by dynamic light scattering (DLS) and electrophoretic light scattering (ELS) measurements. The hydrodynamic diameter (*d*_H_) and polydispersity index (PDI) were reported from the size-intensity distribution function as a mean value of six measurements, while zeta potential (*ζ*) results are expressed as the mean value of three measurements. All measurements were performed at room temperature with PNP concentration of 10 mg/L in ultrapure water (UPW) and in the cell culture medium (CCM) used for further experiments using Zetasizer Ultra (Malvern Panalytical, Malvern, UK) and data were processed using compatible software, ZS Xplorer 3.21 (Malvern Panalytical, Malvern, UK). The CCM consisted of MEM without phenol red (Gibco, Grand Island, NY, USA) and 10% (v/v) charcoal stripped FBS (Sigma Aldrich, Steinheim, Germany). In order to assess colloidal stability of PNPs in CCM, *d*_H_ and *ζ* potential were evaluated immediately upon the dilution and after 24 h. The concentration of 10 mg/L was selected to ensure sufficient signal-to-noise ratio and comparability across particle types under standardized measurement conditions.

Scanning electron microscopy (SEM) was used confirm the size provided by manufacturer and to determine the shape of the PNPs. The samples were analyzed using a High-Vacuum Conventional Scanning Electron Microscope, Inspect F50 (Thermo Fisher Scientific) at various beam voltages and magnifications to obtain a detailed analysis of their morphology and size. Stock concentration of all PNPs was used for analysis, without further dilution to maximize particle visibility and minimize artefacts associated with dilution, enabling reliable assessment of particle morphology. To ensure high-quality imaging and prevent charge buildup on the sample surface, semi-conductive silicon substrates were used. The substrates were thoroughly cleaned by rinsing with ethanol and ultrasonic bath. Once cleaned, the surfaces were allowed to dry completely. A few microliters of the sample were then deposited onto the silicon surface and left to dry in a clean environment overnight. To prevent charge accumulation, particularly as the PNPs are non-conductive, a conductive palladium coating was applied using a sputtering machine. This process deposited a thin, uniform layer of palladium onto the sample surface, ensuring optimal imaging conditions for the SEM analysis.

For individual PNPs experiments, stocks were diluted into CCM to the desired working concentrations and equilibrated for 30 min prior to starting cell treatment. For PNPs mixtures, each particle type was first diluted in CCM to the same per-component concentration; the resulting dispersions were combined (equal volumes) to form the mixture and equilibrated for 30 min before application, ensuring identical final concentrations of each component. For clarity, mixtures are denoted based on the nominal size of their constituent particles, i.e. MIX50 for mixtures containing PS50, PE50, PP50 and PET80, MIX150 for mixtures containing PS150 and PP180, and MIX350 for mixtures containing PS350 and PE350. All mixtures consisted of equal concentrations of each PNP component.

### Exposure concentrations and mixture preparation

PNPs were tested across concentration ranges selected to comply with OECD test guidelines (TGs) and to ensure non-cytotoxic exposure conditions. Therefore, concentration ranges differed slightly between assays due to OECD TGs requirements and assay-specific performance criteria. For ERα and AR transactivation assays (OECD TG 455 and TG 458), concentrations of 0.1, 1, 5, and 10 mg L⁻¹ were used. For the H295R steroidogenesis assay (OECD TG 456), concentrations ranged from 0.25 to 10 mg L⁻¹ to account for assay sensitivity and analytical constraints.

For mixture experiments, each PNP type was first diluted in CCM to the target per-component concentration and then combined in equal volumes. Final concentrations refer to the total nanoparticle mass concentration, with each component contributing equally (e.g., in a four-component mixture at 10 mg L⁻¹ total concentration, each nanoparticle contributes 2.5 mg L⁻¹).

Although slightly different concentration ranges were applied across assays, all exposures across assays were within a non-cytotoxic range and overlapped sufficiently to allow cross-endpoint comparison.

### Cell cultures

The AR-EcoScreen GR KO M1 cell line was purchased from Japanese Collection of Research Bioresources Cell Bank (JCRB Cell Bank, Osaka, Japan) and maintained according to JCRB maintenance protocol. Cells were cultured in tissue culture treated T75 flasks (Sarstedt, Nümbrecht, Germany) and grown at 37 °C and 5% CO_2_ until approximately 90% confluency at which point they were ready for experiments. DMEM/F-12 (Gibco) supplemented with 10% (v/v) fetal bovine serum (FBS, Sigma Aldrich), hygromycin (25 µg/mL, InvivoGen, San Diego, CA, USA) and zeocin (50 µg/mL, InvivoGen, San Diego, CA, USA) was used for cell propagation. The AR-EcoScreen GR KO M1 cell line is a human-derived reporter cell line engineered for androgen receptor transactivation, with glucocorticoid receptor knockout to improve assay specificity.

The HeLa-9903 cell line (JCRB Cell Bank, Osaka, Japan) was maintained in MEM (Gibco, Grand Island, NY, USA) supplemented with 10% (v/v) charcoal stripped FBS (Sigma Aldrich, Steinheim, Germany), 60 µg/mL kanamycin sulphate (Sigma Aldrich, Steinheim, Germany) and 1% L-glutamine (Sigma Aldrich, Steinheim, Germany) until reaching 90% confluency. Cells were cultured in tissue culture treated T75 flasks and grown at 37 °C and 5% CO_2_. The HeLa-9903 cell line is a human cervical carcinoma-derived reporter cell line stably expressing estrogen receptor α used for ER transactivation assays.

The NCI-H295R cell line (American Type Culture Collection, ATCC) was maintained in DMEM/F-12 (Sigma Aldrich) supplemented with 10% (v/v) FBS, 1% (v/v) penicillin-streptomycin (Sigma Aldrich) and 1% (v/v) ITS+ Premix (Corning, Sommerville, USA). Cells were cultured in T75 flasks at 37 °C and 5% CO_2_ for 4 passages before experiment. Cells were used for experiments when they reached 80–90% confluency. The NCI-H295R cell line is a human adrenocortical carcinoma model widely used for assessing steroidogenesis.

### Evaluation of cytotoxicity

MTS assay (Promega, Madison, WI, USA) was used to determine the cytotoxic effects of PNP and their mixtures on AR-EcoScreen, HeLa-9903 and NCI-H295R cell lines. Cells were seeded in clear flat bottom 96-well plates (Eppendorf, Hamburg, Germany) at a density of 4 × 10^4^ cells per well in 100 µL of cell culture media and incubated for 24 h at 37 °C and 5% CO_2_. These cells were then exposed for 24 h to different concentrations of PNPs alone or their mixtures. Non-treated cells were used as negative control, while cells treated with 10% (v/v) DMSO were used as a positive control to confirm assay sensitivity to cytotoxic effects.

### AR transactivation assay

The AR-reporter gene assay was carried out using AR-EcoScreen GR KO M1 cell line in accordance with the OECD TG 458. The cells were seeded in white opaque flat-bottom 96-well plates (Thermo Fisher Scientific, Waltham, MA, USA) at a density of 1 × 10^4^ cells per well and incubated for 24 h before treatment. To reduce interferences, the cells were seeded in a medium consisting of DMEM/F-12 without phenol red supplemented with 5% (v/v) charcoal stripped FBS, hygromycin (25 µg/mL) and zeocin (50 µg/mL). After incubation, the cells were exposed for 24 h to 10, 5, 1 or 0.1 mg/L PNPs alone or their mixtures. Cells treated with dihydrotestosterone (DHT) were used as a positive control, while non-treated cells were used as a negative control. In case of antagonist detection all test chemicals were tested in the presence of DHT and hydroxyflutamide (HF) was applied as AR antagonist control. Following 24 h exposure, the CCM was removed and the cells were washed carefully with PBS. The cell lysate was prepared as suggested in the protocol for Promega luciferase assay system (E1500, Promega, Madison, WI, USA) by adding 20 µL of cell culture lysis reagent diluted 5 times with distilled water. After a 20-minute centrifugation at 300 rpm, the luminescence intensity was measured using SpectraMax iD3 (Molecular Devices, San Jose, CA, USA). Luciferase assay reagent was added with injector system in volume of 100 µL per well. Assay performance and reproducibility were consistent with OECD guideline expectations, with low variability observed across independent experiments.

### ER-α transactivation assay

HeLa-9903 cell line was used to determine ER agonists and antagonists in accordance with OECD TG 455. Cells were seeded in white opaque flat-bottom 96-well plates at a density of 1 × 10^4^ cells/100 µL/well and incubated for 3 h at 37 °C and 5% CO_2_. Cells were exposed for 24 h to individual PNPs or their mixtures at 0.1–10 mg/L.

β-estradiol (E2) was used as positive control and 4-hydroxytamoxifen (OHT) as ERα antagonist. Following that, all test PNPs alone and in mixtures as well as the control substances were added in volume of 50 µL and incubated for 24 h. Afterwards, luciferase activity was measured as described above for the AR-reporter gene assay. Performance and reproducibility of this assay were also consistent with OECD guideline expectations (i.e. <20% for inter- and intra-assay variability).

### Steroidogenesis assay

NCI-H295R cell line was used to assess the impact of PNPs on sex steroid hormone biosynthesis. Cells were seeded in clear flat 24-well plates (Eppendorf) at a density of 2 × 10^5^ cells per well in 1 mL of cell culture media and incubated for 24 h at 37 °C and 5% CO_2_. After incubation, the cells were exposed for 48 h to PNPs alone or their mixtures at concentrations of 0.2–10 mg/L. Besides, cells were treated with prochloraz (1 µM, Sigma Aldrich) as a negative control and forskolin (10 µM, Sigma Aldrich) as a positive control. After exposure, CCM was collected and stored at −80 °C until use. LC-MS/MS and MassChrom^®^ Steroids kit (Chromsystems, Gräfelfing, Germany) were used for the quantitative analysis of steroid hormones. Sample preparation was performed according to the manufacturer’s instructions.

### Data analysis

All experiments were performed in triplicates and repeated independently at least twice. Data for receptor activation/inhibition assays were analysed, presented and interpreted according to OECD TG 455 and TG 458 decision criteria. For cytotoxicity evaluation and steroidogenesis assay (data presented in Supplementary Materials), statistical analysis was done using GraphPad Prism6 (GraphPad Software, San Diego, CA, USA). Statistical significance was assessed using one-way ANOVA followed by Dunnett’s test (*p* < 0.05). For cytotoxicity and steroidogenesis endpoints, significant differences are indicated by an asterisk (*). For ERα and AR transactivation assays, data were interpreted according to OECD TG 455 and TG 458 decision criteria, where biological thresholds rather than statistical significance define activity.

## Results and discussion

### Characterization of PNPs

**Fig. 1:**
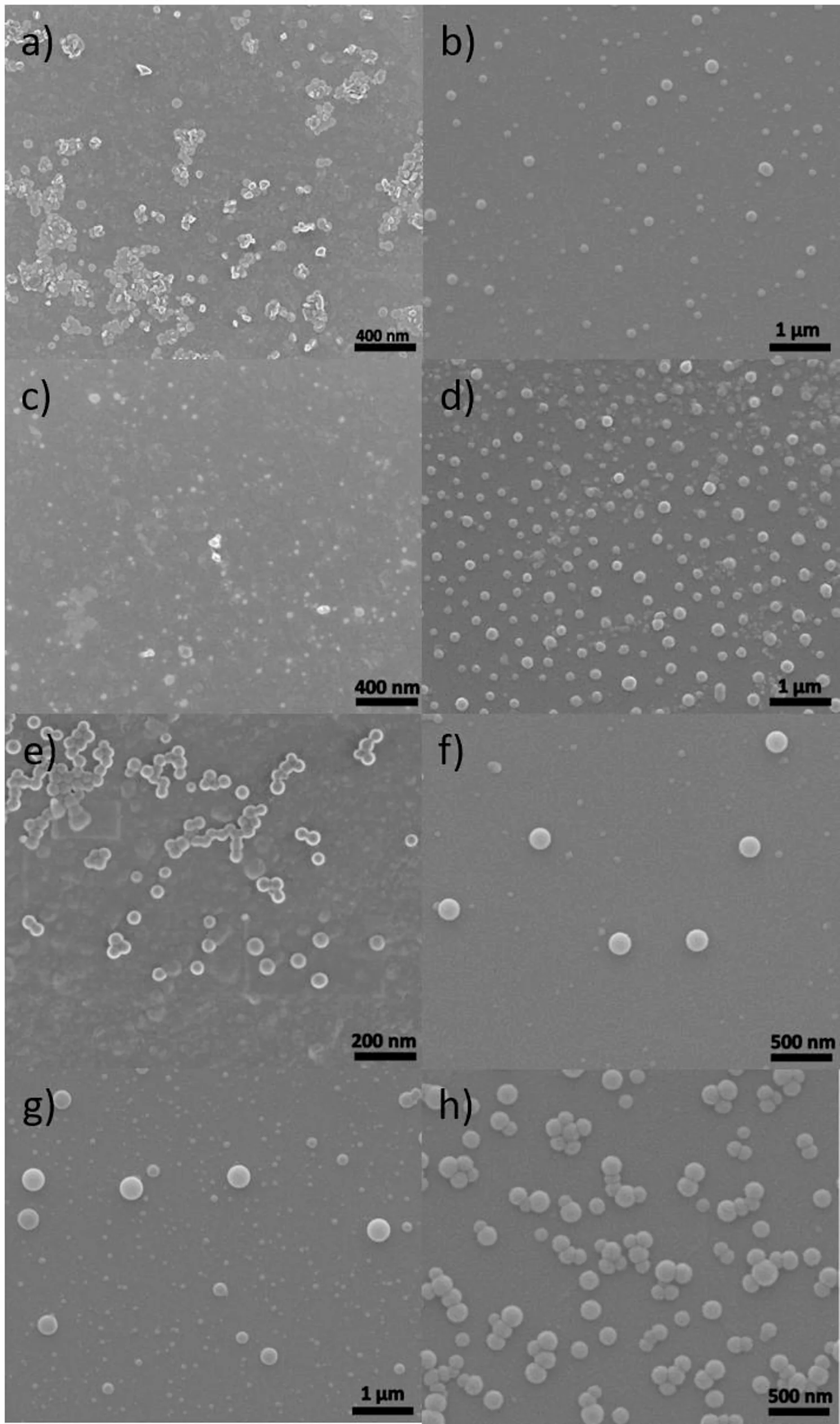
Scanning electron micrographs of PE50 **a**, PE350 **b**, PP50 **c**, PP180 **d**, PS50 **e**, PS150 **f**, PS350 **g** and PET80 **h**. Images were obtained from stock suspensions of PNPs

The morphology of PNPs was investigated using SEM technique and the resulting micrographs are shown in Fig. 1.

All PNPs except PE50 and PP50 were found to be spherical in their shape. The irregular morphology observed for PE50 and PP50 likely reflects differences in particle production routes and polymer physicochemical properties. The semi-crystalline nature and lower glass transition temperatures of PE and PP can favor deformation and irregular morphology during particle formation and drying processes.

Particle size measurements in UPW were performed to compare our results with respect to size in manufacturer’s certificate of analysis. As shown in Table 1, particle size in UPW correlated quite well with the values provided by manufacturer.

**Table 1:** Physico-chemical characteristics of PNPs: density (ρ, g/mL), *ζ* potential (mV) measured by ELS and hydrodynamic diameter (*d*_*H*_, nm) and PDI values determined by DLS

Nanoparticle type	UPW, t = 0			
	ρ (g/mL)	d_H_ (nm)	ζ (mV)	PDI
PS50	1.05	47.3 ± 0.7	−6.1 ± 0.2	0.1
PS150	1.05	208.4 ± 6.6	−5.2 ± 1.2	0.2
PS350	1.05	401.4 ± 29.5	−42.8 ± 1.1	0.3
PE50	0.92	62.5 ± 0.8	−52.7 ± 1.0	0.1
PE350	0.96	351.1 ± 14.2	−32.6 ± 1.6	0.2
PP50	0.90	53.7 ± 0.9	−43.4 ± 2.9	0.2
PP180	0.90	186 ± 1.7	−31.1 ± 0.5	0.1
PET80	1.40	86.5 ± 0.9	−62.4 ± 0.8	0.1

Measurements were conducted at room temperature and the concentration of PNPs was 10 mg/L. Analysis was performed immediately upon preparing the suspension in the UPW

Zeta potential of all PNPs, except PS50 and PS150, is below − 30 mV indicating colloidal stability [35]. To better assess the behavior of nanoparticles during cell treatment, the *d*_H_ and *ζ* potential were also measured in CCM (MEM without phenol red + 5% charcoal-stripped FBS) and results are shown in Table 2.

**Table 2:** Physico-chemical characteristics of PNPs and their mixtures determined by DLS and ELS measurement: *d*_H_ (in nm), *ζ* (in mV) and PDI values

Nanoparticle type	CCM, t = 0			CCM, t = 24 h		
	d_H_ (nm)	ζ (mV)	PDI	d_H_ (nm)	ζ (mV)	PDI
PS50	58.5 ± 6.4	−11.5 ± 1.6	0.3	56.0 ± 6.2	−8.0 ± 0.8	0.4
PS150	483.1 ± 34.6	−10.0 ± 0.3	0.9	451.7 ± 36.7	−9.5 ± 1.8	0.5
PS350	774.2 ± 66.0	−9.9 ± 0.6	0.7	702.5 ± 35.0	−7.1 ± 0.5	0.7
PE50	81.1 ± 5.0	−10.4 ± 2.2	0.5	86.0 ± 2.5	−8.4 ± 0.8	0.5
PE350	377.4 ± 11.7	−16.4 ± 1.2	0.6	377.7 ± 7.1	−16.2 ± 1.3	0.6
PP50	67.5 ± 5.2	−6.4 ± 2.2	0.5	113.2 ± 20.4	−3.4 ± 1.6	0.7
PP180	226.4 ± 7.8	−14.0 ± 0.4	0.2	225.2 ± 4.1	−12.8 ± 1.5	0.2
PET80	121.9 ± 3.9	−11.7 ± 0.4	0.3	119.0 ± 6.3	−7.3 ± 0.9	0.3
MIX 50	119.6 ± 1.5	−13.3 ± 0.7	0.2	110.7 ± 2.1	−13.3 ± 2.4	0.2
MIX 150	192.6 ± 18.0	−11.4 ± 1.1	0.2	193.7 ± 8.9	−12.4 ± 0.6	0.2
MIX 350	354.5 ± 33.6	−13.7 ± 1.1	0.6	665.5 ± 35.3	−16.2 ± 0.6	0.6

Measurements were conducted at room temperature and the concentration of nanoparticles was 10 mg/L. PNPs were diluted in cell culture media and measurements were performed immediately after preparing the suspension and 24 h after

Since the size, shape, surface charge, and surface functionalization of nanoparticles influence their interactions with cells, it is important to study their properties in biological media [36]. Size and ζ stability of single and mixed PNPs was assessed by DLS and ELS measurements immediately after dilution in CCM and 24 h after incubation at 37 °C to mimic cell exposure conditions (Table 2). Increases in *d*_H_ observed in CCM are primarily attributed to protein corona formation. Evidence of aggregation was limited and only observed for selected particles (e.g., PP50), while most particles remained relatively stable over time.

Mixtures were prepared by diluting all PNPs to final concentration of 10 ppm. Obtained results (Table 2) show that the *d*_H_ values in CCM are higher compared to those in UPW. This increase can be attributed to the formation of a protein corona on the surface of PNPs [37]. Additionally, except in the case of 50 nm-sized PPNPs and the mixture of 350 nm-sized PNPs (MIX350), no significant changes in particle diameter were observed after 24 h, indicating stability in the CCM. For PP50 and MIX350, the results suggest aggregation over the 24 h period.

### Evaluation of cytotoxicity

Prior to assessing luciferase activity and performing steroidogenesis assay, the potential cytotoxic effect of individual PNPs and their mixtures was evaluated with MTS assay. A literature review confirmed that the MTS assay is a commonly used method for assessing the cytotoxicity of PNPs [38]. Cytotoxicity of selected concentrations was assessed for both cell lines used as receptor models and for NCI-H295R cell line. Cells were treated with 10, 5, 1 or 0.1 mg/L of individual PNPs or with mixtures prepared by combining each PNP type at equal concentrations. MTS results for both AR-EcoScreen GR KO M1 and HeLa-9903 cell line (Figs. S1 and S2 in Supporting Material) indicate that the individual PNPs at tested concentrations were not cytotoxic. These findings are not unexpected and already demonstrated for PSNPs up to 100 mg/L [39]. Similar results were reported for cytotoxicity effects of PENPs in 9 different cell lines at concentrations up to 200 mg/L, with no significant decrease in cell viability found [40]. Furthermore, PET80 at a concentration up to 125 mg/L were not toxic to A549 cells [41], nor were PPNPs at a concentration of up to 80 mg/L to Caco-2 cells [42]. Our results demonstrated no cytotoxic effects observed across all tested concentrations and PNP types, even when combined in the various mixtures, in HeLa-9903 and AR-EcoScreen GR KO M1 cells (Figure S3 in Supporting Material). Similar results were obtained for NCI-H295R cells (Figure S4 in Supporting Material). Based on these results, subsequent experiments were planned with non-toxic doses of PNPs. Quantification of cellular uptake and internal dose was beyond the scope of this study; however, future work integrating quantitative dosimetry approaches will be essential to refine exposure-response relationships and support *in vitro/in vivo* extrapolation.

### AR and ER-α transactivation assays

**Fig. 2:**
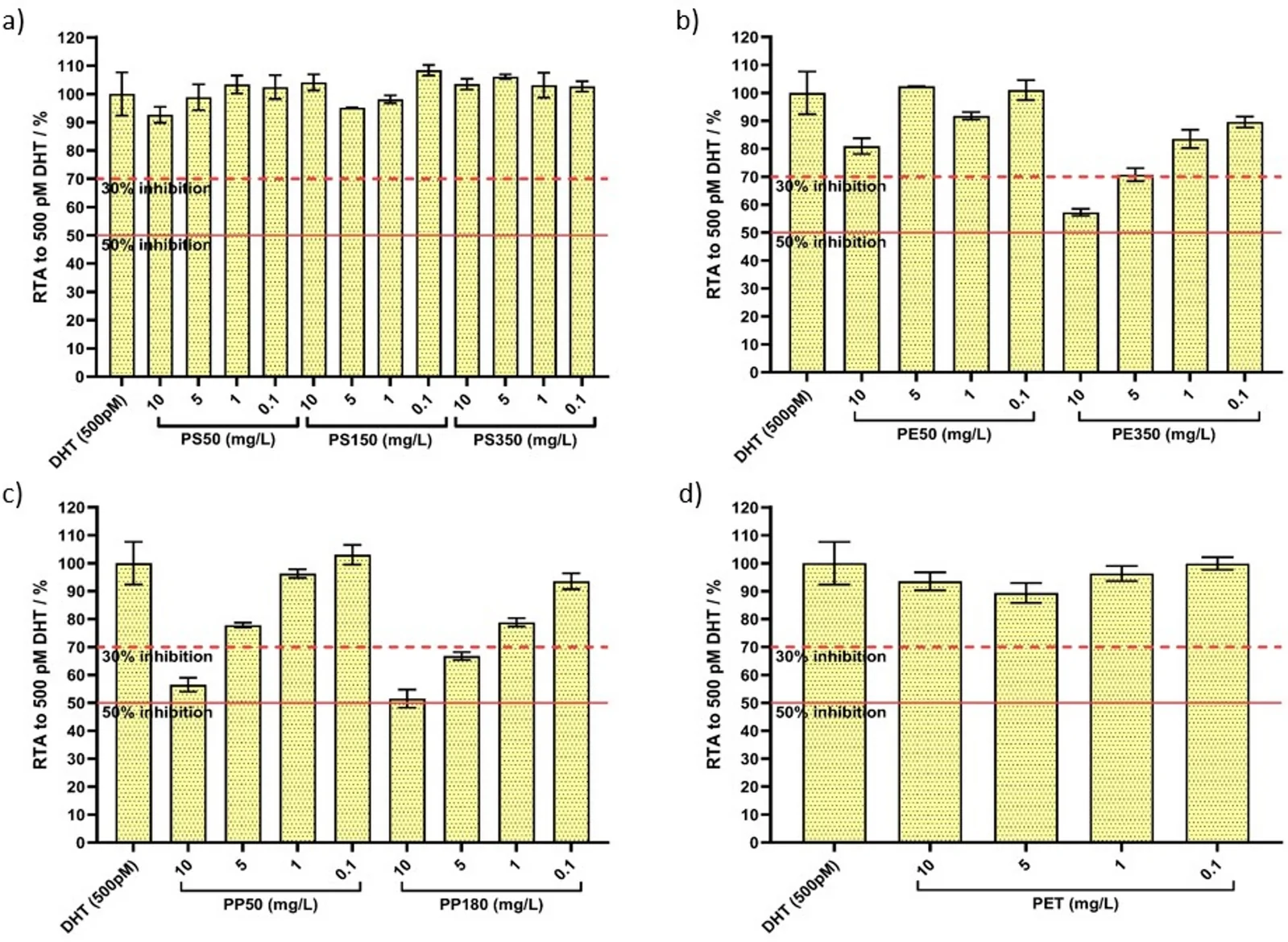
AR antagonist effect of **a** PS50, PS150, PS350, **b** PE50, PE350, **c** PP50, PP180, and **d** PET80. Results are showed as relative transcriptional activity compared to 500 pM DHT (agonist reference). Data are expressed as mean value of replicates from two repeated experiments and standard deviations are given as error bars

**Fig. 3:**
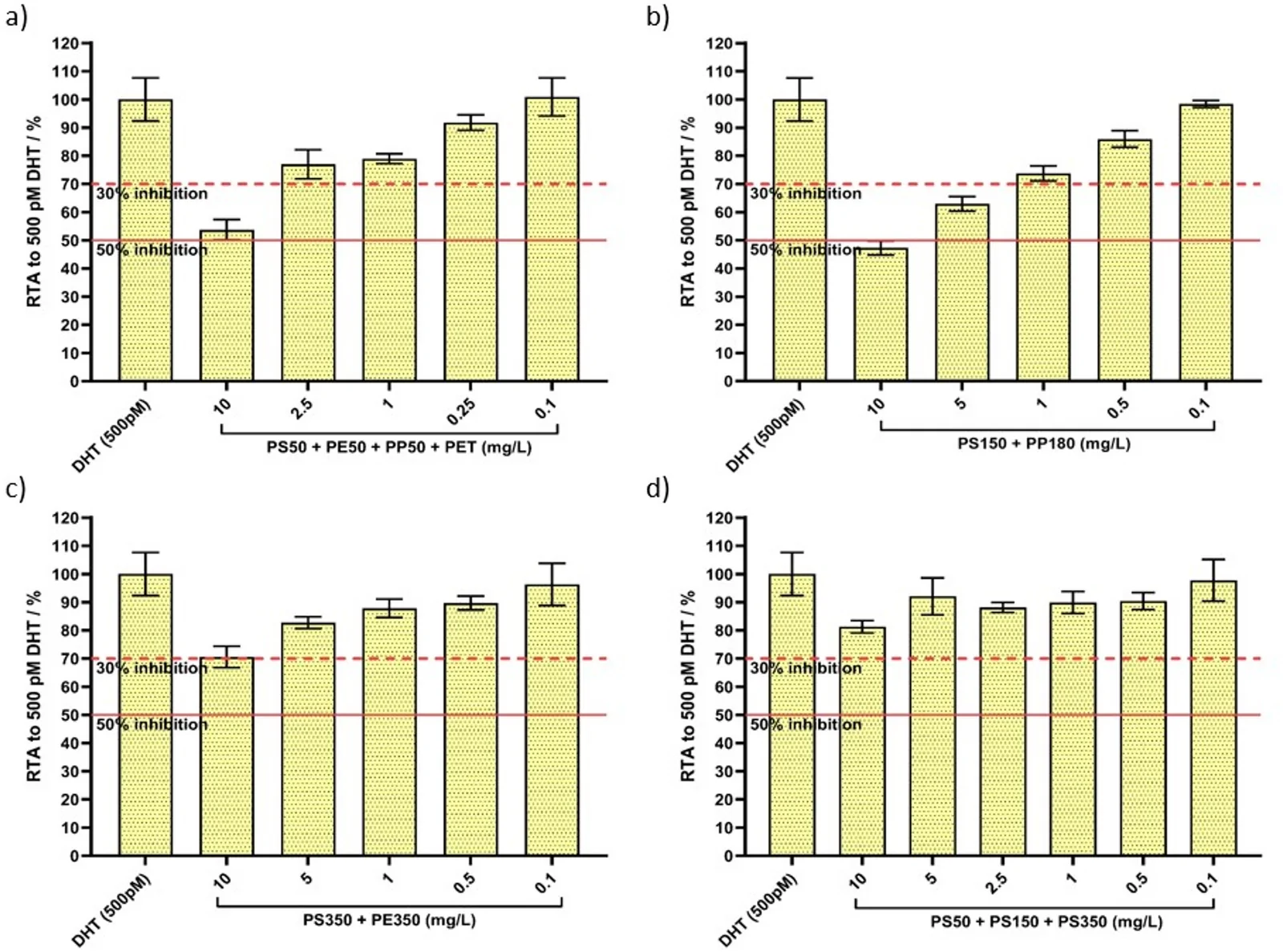
AR antagonist effect of **a** mixture of PS50, PE50, PP50 and PET80, **b** mixture of PS150 and PP180, **c** mixture of PS350 and PE350, **d** mixture of PS50, PS150 and PS350. Mixtures were prepared by combining each nanoparticle type in the same concentration. Results are showed as relative transcriptional activity compared to 500 pM DHT (agonist reference). Data are expressed as mean value of replicates from two repeated experiments and standard deviations are given as error bars

The AR antagonistic activity of PNPs, both individually and in mixtures, was tested following OECD TG 458, but using AR-EcoScreen GR KO M1 cells instead of AR-EcoScreen cell line recommended by TG. The AR-EcoScreen GR KO M1 cell line provides higher accuracy in determining (anti-)androgens in comparison with AR-EcoScreen because of glucocorticoid receptor gene knockout [43]. Decision criteria of TG 458 for AR antagonist assay is that chemical should inhibit response of 500 pM DHT by at least 30%. Based on this, only PE350, PP50 and PP180 alone were found to have AR antagonist activity (Fig. 2). Regardless of the particle size, PSNPs and PET80 did not exhibit AR antagonistic activity (Fig. 2a and d) at any tested concentration (10, 5, 1, and 0.1 mg/L). These results align with a recent study on the potential AR antagonistic effects of PS-based PMPs and PNPs [44]. On the contrary, results for PENPs were dependent on the size and concentration with PE350 to be considered AR antagonists in a dose-dependent manner (Fig. 2b). Similar dose- and size-dependent effects were observed for PPNPs. Higher doses induced more pronounced inhibition of DHT response with larger particles being stronger AR antagonists (Fig. 2c). Results for PNPs mixtures are presented in Fig. 3. The MIX50 and MIX150 strongly inhibited the DHT-induced response, and again larger particles (MIX150) reduced it to higher extent (by over 50%).

These results are consistent with the dose-response inhibition of DHT response inhibition observed for individual PNPs. Results for PSNPs mixture were expected following observations for individual PSNPs, as this mixture did not meet the threshold for AR antagonistic activity at any of the tested concentrations. In the case of MIX350, treatment with the highest dose (10 mg/L) was on the borderline to be considered as AR antagonistic, while lower concentrations did not show effects (Fig. 2c).

The divergent behavior of PP/PE vs. PS/PET PNPs suggests a surface-property–driven mechanism rather than a generic particle effect. Compared with PS/PET, PP and PE present more hydrophobic, less aromatic surfaces and (in aqueous protein-containing media) tend to form distinct coronas that can alter interactions with the AR signaling axis [45]. The inactivity of PS/PET is consistent with weaker DHT partitioning and/or coronas that are less disruptive to AR signaling under these conditions. Moreover, the stronger AR antagonism by larger PP180 vs. PP50, despite smaller particles having higher specific surface area, implies that curvature-dependent (i.e., particle size-dependent surface geometry) corona composition and effective local concentration dominate over simple area scaling (i.e., assuming effects scale only with particle surface area). Larger particles can stabilize coronas with higher AR-relevant protein content, sediment/settle into closer proximity with cells, increasing the particle–cell contact time in static cultures, and/or reduce DHT diffusion at the cell interface (unstirred-layer effects). For PENPs, a non-monotonic size–activity relation (PE150 > PE50, and PE350 meeting the OECD-defined activity threshold) is compatible with competing processes, e.g., stronger DHT scavenging at certain curvature/corona states counterbalanced by differences in particle number and transport [46–48].

The enhancement in MIX50/MIX150 relative to singles indicates additive or supra-additive antagonism when PPNPs and PENPs co-occur, perhaps via broader sequestration of DHT (wider sorption domain across polymers/sizes) and/or complementary corona milieus that more effectively dampen AR signaling. The near-threshold response of MIX350 at only the higher dose suggests that at higher mass loadings diffusion/partitioning limits dominate, while at lower doses pharmacodynamic interference alone is insufficient [49].

**Fig. 4:**
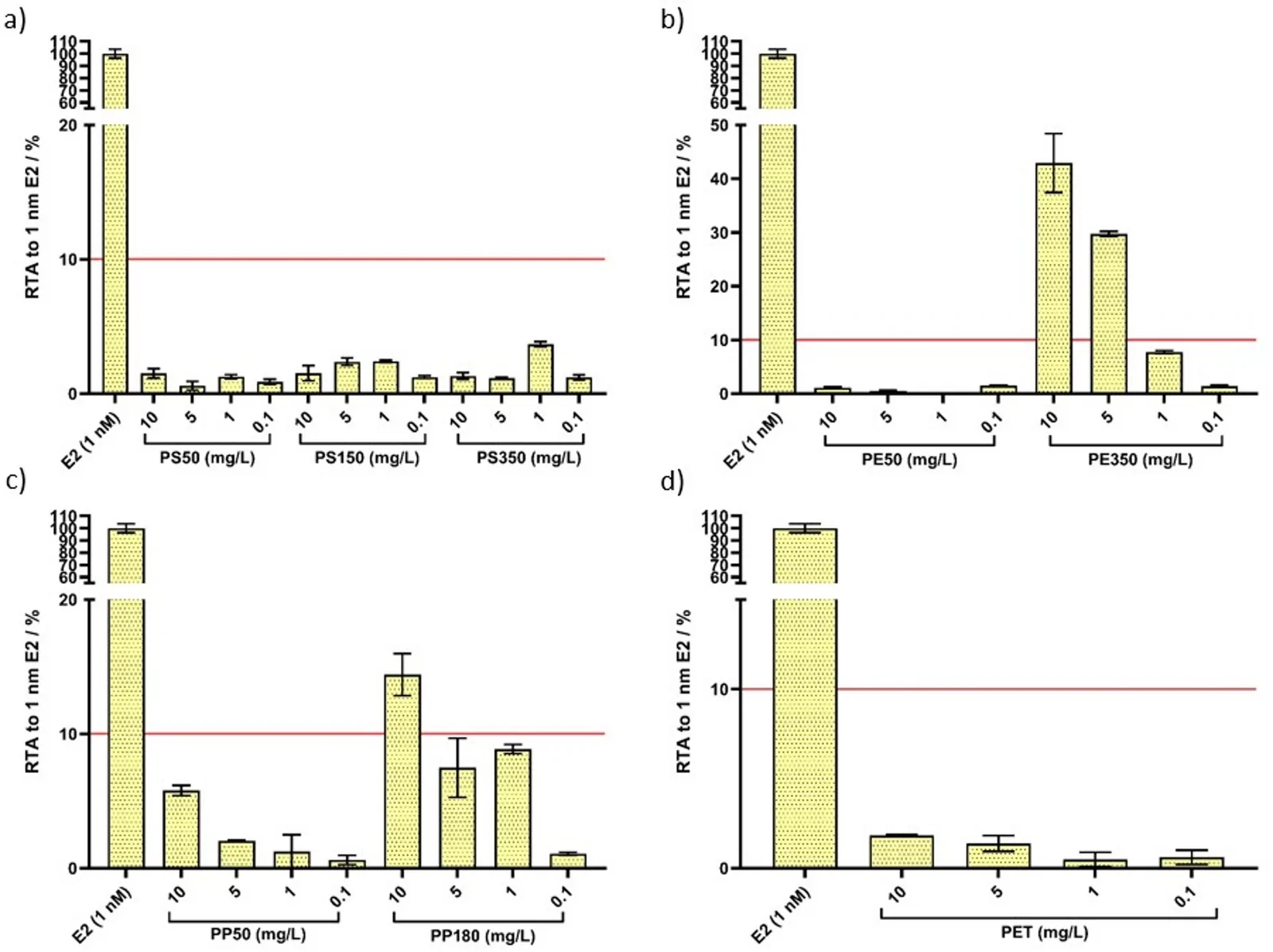
ER agonist effect of **a** PS50, PS150, PS350, **b** PE50, PE350, **c** PP50, PP180, **d** PET80. Results are showed as relative transcriptional activity compared to 1 nm E2 (positive control). Data are expressed as mean value of replicates from two repeated experiments and standard deviations are given as error bars

The assessment of the ER agonistic activity of PNPs was conducted according to OECD TG 455. The criterion for identifying ER agonists is achieved if at least 10% of the response induced by E2 (positive control) is reached. As shown in Fig. 4, this was observed only for PP180 and PE350, while all other PNPs did not demonstrate ER agonistic activity at any of tested concentrations.

The inactivity of PS across sizes aligns with recent study on VM7 cell line showing that PS-MNPs of sizes between 50 nm and 10 μm and in concentration range 0.01–100 mg/L do not activate ER under comparable reporter conditions, [44] and is consistent with our prior T47D-KBluc observations that ER activity is polymer-specific, emerging for PE/PP but not PS [25]. This selectivity indicates that ER-mediated responses are not a generic particle effect but depend on polymer chemistry and particle size. As in the case of results obtained for AR antagonistic effects (Fig. 3), the same rationale can be used here. The PE/PP present highly hydrophobic, non-aromatic surfaces that, in serum-containing media, acquire distinct protein/lipid coronas, which may modulate ligand access to receptors, co-regulator recruitment, and nuclear translocation [45]. Moreover, obtained ER agonistic acitivity for different sizes of PPNPs and PENPs (PP180 > PP50 and PE350 > PE50) points to curvature-dependent corona composition and cell-layer dosimetry (greater effective contact dose in static cultures due to settling), rather than a simple explanation that higher specific surface area exhibit more pronounced more effect [49].

**Fig. 5:**
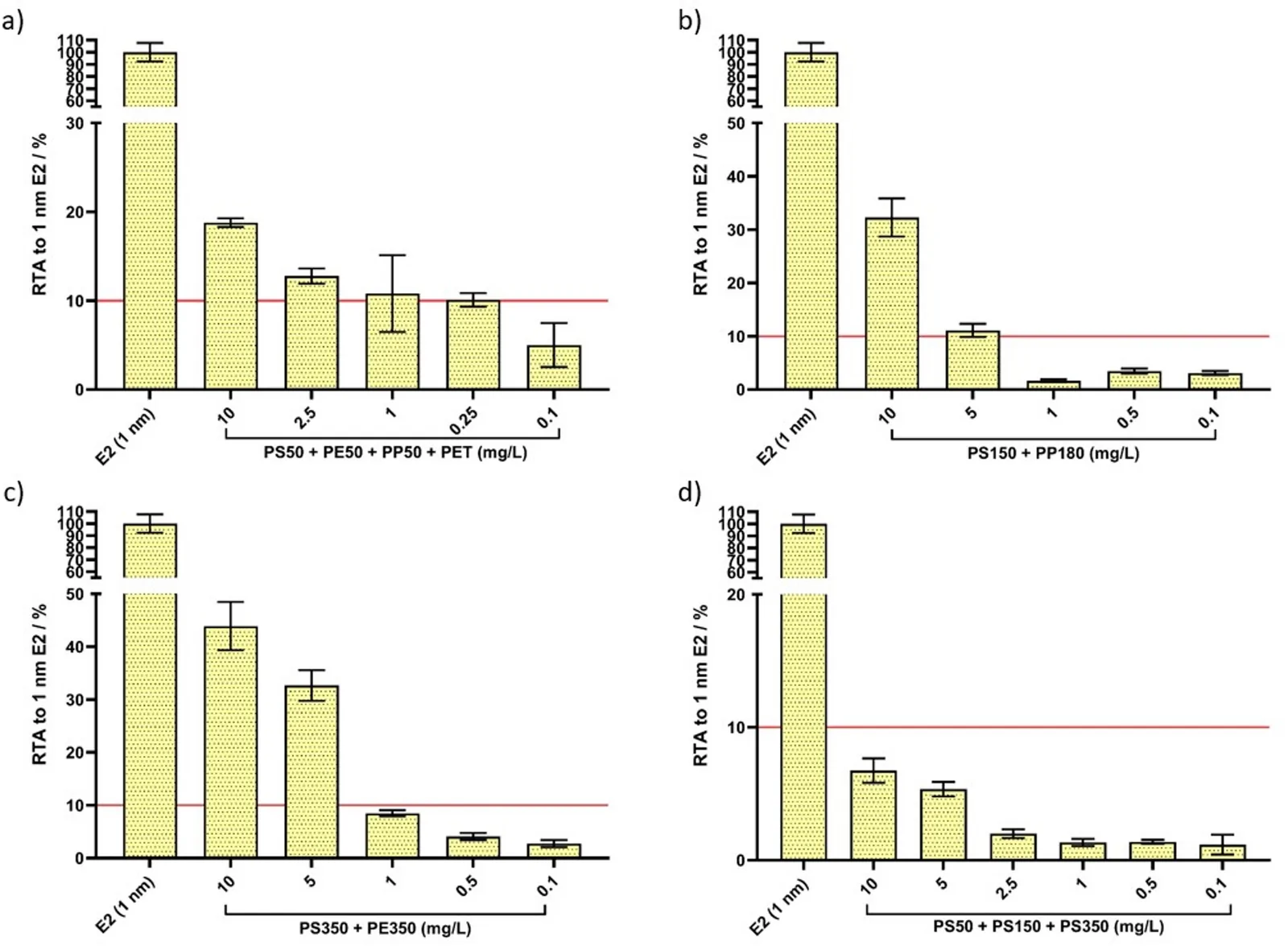
ER agonist effect of **a** mixture of PS50, PE50, PP50 and PET, **b** mixture of PS150 and PP180, **c** mixture of PS350 and PE350, **d** mixture of PS50, PS150 and PS350. Mixtures were prepared by combining each nanoparticle type in the same concentration. Results are showed as relative transcriptional activity compared to 1 nm E2 (positive control). Data are expressed as mean value of replicates from two repeated experiments and standard deviations are given as error bars

ER activation following treatment with different PNPs mixtures is presented in Fig. 5. It is evident that the highest concentration of all mixtures, except for PSNPs mixture, exceeds the 10% threshold. This pattern is consistent with concentration addition/response additivity among weak ER-active inputs and/or mixture-altered coronas that enhance ER signaling efficiency [50]. The PS-only mixture mirrored the inactivity of its constituents, reinforcing a polymer-specific driver.

In both data sets, PE and PP nanoplastics induced activity: ER agonism for PE350, PP180 and AR antagonism for PE350, PP50, PP180, whereas PSNPs in all sizes and PET80 were inactive. Activity was generally stronger around ~ 150–350 nm, and polymer-matched mixtures outperformed most singles. Taken together, these findings suggest a receptor-proximal mode of action driven by surface properties and particle–cell interactions. However, it should be noted that AR antagonism was measured in the presence of a fixed DHT stimulus at 500 pM. Any process that reduces free DHT at the cell surface (e.g., partitioning/adsorption into PNPs corona complexes, or corona-mediated receptor/co-regulator interference) may lower the AR signal. On the other hand, ER agonism was measured without exogenous E2 and response is expressed vs. 1 nM E2 stimulus as positive control. In this assay, the same nanosurface and/or biomolecular corona features can yield net reporter activation through corona-driven changes in receptor/co-regulator availability or membrane-initiated ER signaling. As E2 was not added in wells with PNPs treatments, ligand scavenging cannot depress an agonist signal.

The greater activity of PP180 vs. PP50 and PE350 vs. PE50 across two assays suggests curvature-dependent corona composition and dominant cell-layer dosimetry over simple arguments related to the specific surface area. Under static in vitro conditions, particle transport to the cell layer is influenced by sedimentation and diffusion processes, which contribute to the effective cellular dose. However, such behavior does not directly reflect in vivo conditions, where particles are subject to dynamic flow, shear forces, and complex biological interactions. Therefore, sedimentation effects should be interpreted as a feature of in vitro exposure systems rather than a direct predictor of in vivo behavior. The mixture enhancement, especially for MIX150, further implies complementary coronas and a wider sorption domain across sizes/polymers, amplifying both effects.

Finally, impact of particle density should be also considered as this PNP characteristic determines also delivered cell dose. The densities of tested PNP (Table 1) span sub-, near-, and supra-aqueous values. PP (0.90 g/mL) and PE (0.92–0.96 g/mL) particles were less dense than water, PSNPs (1.05 g/mL) were slightly denser, while PETNPs (1.40 g/mL) was substantially denser. In static plates, the delivered dose to the cell layer is controlled by the balance of gravitational settling and Brownian diffusion, which leads to the faster deposition of denser and larger particles at the bottom monolayer than lighter/smaller ones [51]. Thus, PS and PET particles would achieve higher bottom-layer contact than PP/PE under identical hydrodynamic diameters, while the strong size effect on settling means PS350 deposits far faster than PS50, whereas PET80 remains predominantly diffusion-driven despite its density [51]. 

These transport principles strengthen our biological interpretation as strongest AR antagonism/ER agonism was observed for PP/PE (PP50, PP180, PE350) even though their buoyancy biases against gravitational delivery. On the contrary, PS and PET, whose densities favor bottom-layer exposure, were largely inactive when applied alone. This divergence indicates that the observed endocrine activity is not an artifact of over-delivery by settling and instead supports a receptor-proximal, surface/corona mechanism (consistent with the OECD-negative TG 456 result). In practice, three factors can narrow the density gap in culture: (i) biomolecular coronas formed in serum can increase effective density and agglomerate size, accelerating deposition relative to UPW values; [52] (ii) near-wall diffusion and adhesion still bring buoyant particles into cell contact; [51] and (iii) hetero-agglomeration in mixtures can raise both effective density and *d*_H_, enhancing delivered dose, which is consistent with mixture potentiation [51, 52].

To further evaluate whether these receptor-mediated effects could be linked to alterations in steroid hormone biosynthesis, we next assessed steroidogenesis using the OECD TG 456.

### Steroidogenesis assay

**Fig. 6:**
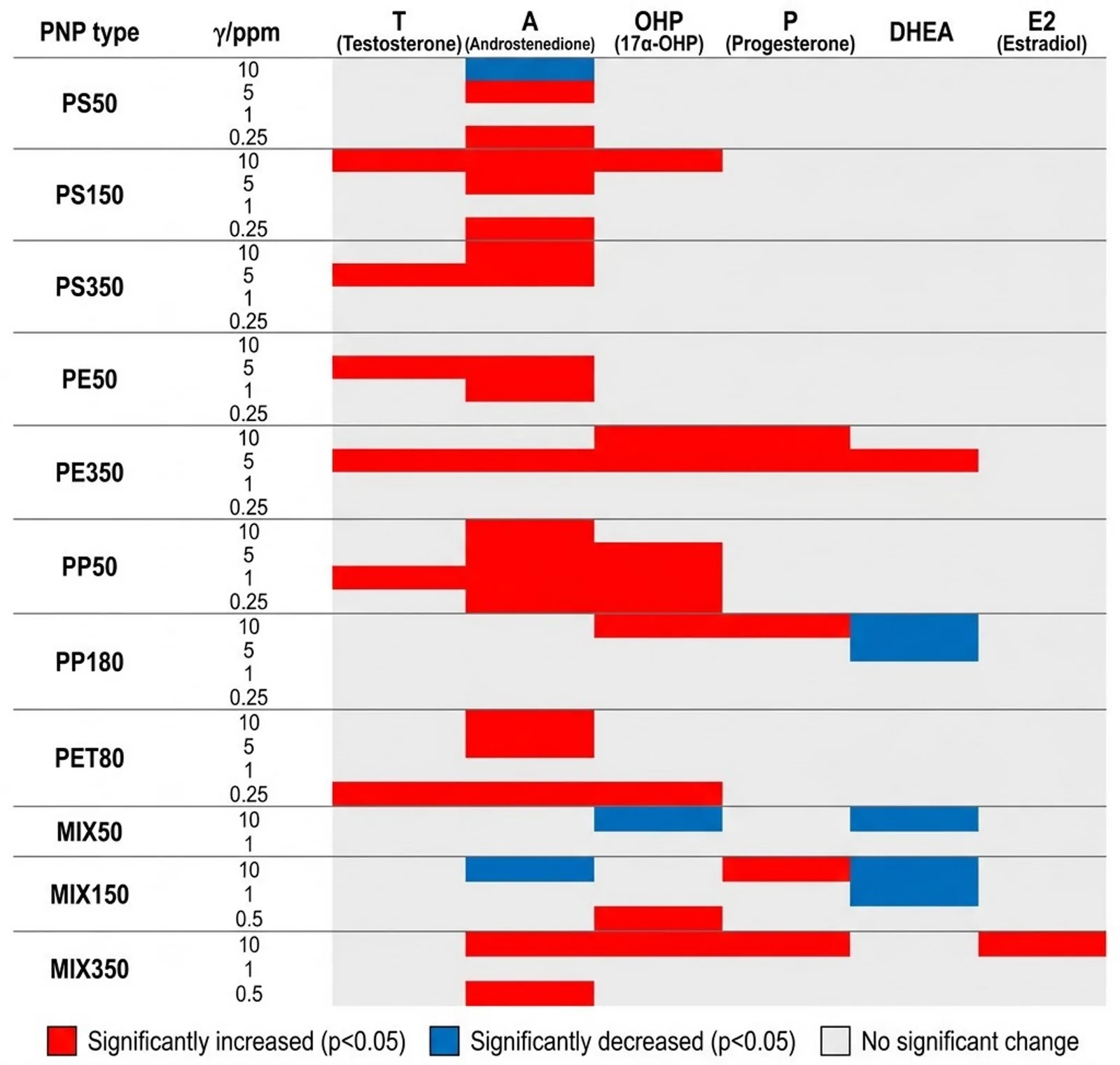
Summary of statistically significant changes in steroid hormone levels (testosterone - T; androstenedione - A; 17α-Hydroxyprogesterone - OHP; progesterone - P; dehydroepiandrosterone - DHEA; estradiol - E2) in H295R cell culture media following exposure to PNPs and their mixtures. Red cells indicate significantly (at *p* < 0.05) increased, blue cell indicate significantly (at *p* < 0.05) decreased hormone levels in treated vs. non-treated cells. Uncolored fields indicate no significant differences in hormone concentration

Steroid hormone outputs were quantified in culture supernatants by LC-MS/MS after 48 h exposure of NCI-H295R cells to size-resolved PS, PE, PP, PET nanoplastics and polymer-matched mixtures (MIX50/MIX150/MIX350) across 0.25–10 mg/L, with viability data collected in parallel to support interpretation (Figures S4–S10 in Supporting Material). Statistical testing used one-way ANOVA with Dunnett’s post hoc comparison to solvent control. Per OECD criteria (positive call requires statistically significant changes in testosterone [T] or estradiol [E2] at two adjacent concentrations in at least two independent runs) [31], none of the individual PNPs or their mixtures met the decision rule (Fig. 6).

Although certain changes in the concentrations of steroidogenic intermediates were observed, these alterations were not accompanied by significant changes in E2 levels, except in the case of the highest concentration of MIX350. In the case of T levels, significant increase was observed for treatments with 10 mg/L of PS150, 5 mg/L of PS350, 5 mg/L of PE50 and PE350, 1 mg/L of PP50 and 0.25 mg/L of PET80. For upstream intermediates (e.g., androstenedione, 17-OH-progesterone, progesterone, dehydroepiandrosterone), only sporadic, dose-specific changes were observed that were not mirrored by concordant changes in E2 or T (Figures S5–S10 in Supporting Material). This pattern suggests partial modulation of individual steroidogenic steps with homeostatic flux buffering within the network, and/or re-routing pathway between the Δ5 (pregnenolone → DHEA → A4) and Δ4 (progesterone → 17-OH-progesterone → A4) branches that preserves terminal hormone output [53]. In enzyme-kinetic terms, small changes in activities with low flux-control coefficients (e.g., 3β-HSD, 17β-HSD, CYP17A1 hydroxylase/lyase split, CYP19) can alter pool sizes of intermediates without overcoming downstream compensatory capacity at aromatase or 17β-HSD [54]. Additionally, substrate transport and cofactor availability (e.g., StAR-mediated cholesterol import, NADPH supply) can impose constraints that dampen propagation of intermediate shifts to E2/T [55]. Finally, particle–analyte interactions such as adsorption/partitioning of lipophilic steroids to PNP surfaces may bias measured intermediate levels in supernatants without materially changing net synthesis of terminal hormones [56]. Together with preserved cell viability (Figure S4), these features are consistent with modest, compensated perturbations that fall short of the OECD TG 456 decision rule.

The present findings can be contextualized with emerging in vivo evidence of endocrine-related effects induced by PMPs and PNPs. Experimental studies in rodent models have shown that exposure to polystyrene micro- and nanoplastics is associated with decreased testosterone levels, impaired spermatogenesis, and reduced sperm quality, ultimately leading to diminished male fertility [18, 19, 58]. For example, oral exposure to PSNP at doses in the range of tens of mg kg⁻¹ body weight has been reported to reduce serum testosterone levels and disrupt testicular function [58, 59]. The influence of PSNPs on steroidogenesis was also investigated in the study by Van Boxel et al., whose results showed that treatment with PSNPs did not significantly affected testosterone or estradiol concentrations, indicating no effect on steroidogenesis [44]. Opposite to that, PSNPs disrupted steroidogenesis in swine granulosa cells by increasing E2 levels and decreasing progesterone levels [57].

While recent in vivo studies predominantly involve PS particles, and often at higher exposure levels than those used in vitro, the observed endocrine-related outcomes are broadly consistent with receptor-mediated perturbations identified in the present study. Importantly, our findings extend this evidence by demonstrating that polymer type and particle size critically influence receptor activity, with PP and PE nanoplastics showing distinct ER and AR modulation, whereas PS remained inactive under the tested conditions. Direct extrapolation between in vitro and in vivo systems remains challenging due to differences in exposure routes, dose metrics (mass concentration vs. body burden), and particle behavior in biological systems. Nevertheless, the receptor-level effects observed here provide a mechanistic basis that may contribute to the endocrine and reproductive alterations reported in vivo, supporting the relevance of nanoplastics as potential endocrine-active materials.

Overall, under the assay conditions used here, PNP exposure did not trigger positive steroidogenic response. The isolated shifts in intermediates - uncoupled from E2/T - may reflect modest, compensated pathway perturbations and/or non-monotonic responses that fall short of the TG 456 decision threshold. Importantly, the absence of a positive outcome in the H295R steroidogenesis assay indicates that the ER and AR activities observed in reporter assays are unlikely to be secondary to altered hormone biosynthesis. Instead, these findings support a receptor-proximal mechanism occurring at the particle–cell interface. When considered together with the polymer- and size-dependent receptor responses and mixture effects described above, the data are consistent with a surface-mediated mode of action.

At the organism level, receptor-mediated perturbations of ER and AR signaling represent early molecular events that may translate into broader endocrine and physiological effects. Disruption of ER and AR pathways is known to influence reproductive function, including gametogenesis, hormone regulation, and fertility, as well as developmental and metabolic processes. In this context, the polymer- and size-dependent modulation of ER and AR activity observed in the present study suggests that nanoplastics may act as endocrine-active materials capable of interfering with hormonal signaling networks. Although the magnitude of effects observed in vitro does not directly predict organism-level outcomes, such receptor-level interactions provide a mechanistic basis for the reproductive and endocrine alterations reported in animal studies following PMPs and PNPs exposure. Importantly, the identification of mixture effects further indicates that combined exposures, more representative of real-world scenarios, may amplify biological responses even when individual components are weakly active. Together, these findings highlight the need to consider nanoplastics not only as inert particles but as biologically interactive materials with potential implications for human health.

## Conclusion and future perspective

Our comparative, OECD-aligned test battery shows that the endocrine activity of nanoplastics is dictated by polymer identity and particle size. Polypropylene (50 and 180 nm) and polyethylene (350 nm) NPs consistently antagonized AR, while polypropylene (180 nm) and polyethylene (350 nm) activated ER. In contrast, polystyrene (50, 150 and 350 nm) and polyethylene terephtalate (80 nm) NPs were inactive as single materials under the same conditions. Mixtures frequently enhanced ER and AR responses relative to singles, mostly where individual components remained sub-threshold, which indicates that mixture testing is essential for environmentally realistic hazard appraisal. At the organism level, such receptor-mediated perturbations may contribute to altered endocrine function, with potential implications for reproductive health and hormonal homeostasis.

Despite occasional shifts in steroidogenic intermediates, the H295R steroidogenesis assay did not meet the OECD decision rule for altered steroidogenesis, indicating that the observed ER and AR signals are unlikely to stem from altered hormone biosynthesis and instead reflect receptor-proximal interactions at the particle-cell interface. Taken together, these findings argue for moving beyond PS-only surrogates in endocrine testing, prioritizing PP, PE and PET nanoplastics for targeted monitoring and mechanistic investigation, alongside systematic inclusion of mixture designs and surface-property characterization.

The observed endocrine activity patterns support a receptor-proximal, surface-mediated mode of action for PNPs. Specifically, the absence of steroidogenic disruption (TG 456 negative outcome) combined with polymer- and size-dependent receptor responses and mixture potentiation, indicates that interactions occur at the particle–cell interface rather than through intracellular endocrine pathway perturbation. A plausible mechanistic basis involves differential biomolecular corona formation across polymers and sizes, which may modulate ligand availability, receptor binding, or co-regulator recruitment. In particular, hydrophobic PP and PE surfaces are expected to form coronas with higher affinity for lipophilic molecules and signaling proteins, potentially altering AR and ER signaling dynamics. While direct corona characterization was beyond the scope of this study, the consistent structure–activity relationships observed here strongly support this mechanism and provide a testable hypothesis for future work.

Several limitations should be considered when interpreting these finding. The use of static in vitro exposure systems means that particle transport to the cell layer is influenced by sedimentation and diffusion. which contribute to effective cellular dose under these conditions but do not directly reflect dynamic in vivo environments such as circulating blood. In physiological systems, PNPs behavior is governed by flow conditions, evolving biomolecular coronas, and tissue-specific distribution, and thus sedimentation-driven delivery may be overestimated in vitro. In addition, quantitative determination of intracellular PNP dose was beyond the scope of this study, limiting precise exposure-response interpretation. Finally, the availability of well-characterized PNPs constrained the size range for non-polystyrene polymers, particularly PET, for which only a single particle size was tested. Together, these factors highlight broader challenges in nanoplastics research and underscore the need for improved reference materials and physiologically relevant exposure models.

Future studies should therefore consolidate mechanistic understanding and strengthen translational relevance. First, pharmacokinetic from pharmacodynamic contributions should be discriminate by performing ligand spike–recovery with PNPs present in assay media to quantify adsorption/partitioning and potential LC–MS/MS matrix effects, as well as by running titrations of agonist with PNPs to differentiate ligand depletion from receptor/co-regulator interference. Second, leachate-driven from surface/corona-driven effects should be separated by testing PNPs extracts alongside washed-PNPs resuspensions and verifying ER specificity with fulvestrant or 4-hydroxytamoxifen, as well as by confirming luciferase results with an orthogonal endpoint (e.g., ERE-qPCR for TFF1/PGR). Finally, lower, environmentally relevant exposures and co-exposures should be evaluated by including polymer- and size-diverse mixtures, and by screening with surface-aware analytics to correctly attribute ER or AR activity to nanoscale interface mechanisms rather than systemic steroidogenic disruption. Such strategies will enable more robust integration of nanoplastics into human health risk assessment frameworks.

## Supplementary Information

Supplementary Material 1.

## Data Availability

The authors confirm that the data supporting the findings of this study are available within the article and its supplementary materials.
