# Supplementary material for: Surface-driven endocrine activity of nanoplastics: polymer- and size-dependent estrogen and androgen receptors modulation without steroidogenesis perturbation
Source: Part Fibre Toxicol. 2026 Jun 10;23:40. doi: 10.1186/s12989-026-00692-7 (PMC13520334; doi:10.1186/s12989-026-00692-7)
Supplement: Supplementary file 1 [file 12989_2026_692_MOESM1_ESM.docx]

**Supplementary Material for:**

**Surface-Driven Endocrine Activity of Nanoplastics: Polymer- and Size-Dependent Estrogen and Androgen Receptors Modulation without Steroidogenesis Perturbation**

Nikolina Peranić,^a^ Lucija Božičević,^a^ Nikolina Kalčec,^a^ Korinna Altmann,^b^ Jana Hildebrandt,^b^ Carlos Cuestas Ayllon,^c^ Raquel Portela,^d^ Jesús M de la Fuente,^c^ Miguel A. Bañares,^d^ Ivana Vinković Vrček ^a,e^

a Institute for Medical Research and Occupational Health, Ksaverska cesta 2, 10000 Zagreb, Croatia

b Bundesanstalt für Materialforschung und prüfung, Unter den Eichen 87, 12205 Berlin, Germany

c Instituto de Nanociencia y Materiales de Aragón, CSIC-Universidad de Zaragoza and CIBER-BBN, c/Pedro Cerbuna s/n, 50009 Zaragoza, Spain

d Institute for Catalysis and Petrochemistry, CSIC, C. de Marie Curie 2, E 28049, Cantoblanco, Madrid, Spain

e University of Rijeka, Faculty of Medicine, Braće Branchetta 20, Rijeka, Croatia

**Figure S1.** Cell viability for AR-EcoScreen GR KO M1 cell line treated with 10, 5, 1 and 0.1 mg/L of: a) PS50, PS150, PS350, b) PE50, PE350, c) PP50, PP180 and d) PET. Cells treated with 10% DMSO were used as positive control (DMSO) and non-treated cells were used as negative control (CTRL). Results are expressed as mean value of replicates from two experiments and standard deviations are given as error bars.

**Figure S2.** Cell viability for HeLa-9903 cell line treated with 10, 5, 1 and 0.1 mg/L of: a) PS50, PS150, PS350, b) PE50, PE350, c) PP50, PP180 and d) PET. Cells treated with 10% DMSO were used as positive control (DMSO) and non-treated cells were used as negative control (CTRL). Results are expressed as mean value of replicates from two experiments and standard deviations are given as error bars.

**Figure S3.** Cell viability for AR-EcoScreen GR KO M1 and HeLa-9903 cell lines treated with: a) 10, 2.5, 1, 0.25 and 0.1 mg/L of PS50, PE50, PP50 and PET, b) 10, 5, 1, 0.5, 0.1 mg/L of PS150 and PP180, c) 10, 5, 1, 0.5, 0.1 mg/L of PS350 and PE350, d) 10, 5, 2.5, 1, 0.5, 0.1 mg/L PS50, P150 and PS350. Mixtures were prepared by combining each nanoparticle type in the same concentration. Cells treated with 10% DMSO were used as positive control (DMSO) and non-treated cells were used as positive control (CTRL). Results are expressed as mean value of replicates from two experiments and standard deviations are given as error bars.

**Figure S4.** Cell viability for NCI-H295R cell line treated with: 10, 5, 1 and 0.1 mg/L of a) PS50, PS150, PS350; b) PE50, PE350; c) PP50, PP180; d) PET; e) 10, 1 mg/L of PS50, PE50, PP50 and PET; 10, 1, 0.5 mg/L of PS150 and PP180; 10, 1, 0.5 mg/L of PS350 and PE350. Mixtures were prepared by combining each nanoparticle type in the same concentration. Cells treated with 10% DMSO were used as positive control (DMSO) and non-treated cells were used as positive control (CTRL). Results are expressed as mean value of replicates from two experiments and standard deviations are given as error bars.

**Figure S5.** Testosterone concentration in NCI-H295R culture media upon exposure to: a) 10, 5, 1 and 0.25 mg/L of PSNP 50 nm, PSNP 150 nm, PSNP 350 nm; b) 10, 5, 1 and 0.25 mg/L PENP 50 nm, PENP 350 nm; c) 10, 5, 1 and 0.25 mg/L PPNP 50 nm, PPNP 180 nm and d) 10, 5, 1, 0.25 mg/L PET, 10 and 1 mg/L MIX50, 10, 1 and 0,5 mg/L MIX150, 10 and 1 mg/L MIX350. Results are expressed as a mean value of triplicates from two individual experiments. Statistically significant changes (* p < 0.05) relative to the negative control were calculated with one-way ANOVA and Dunnett’s multiple comparisons test in GraphPad Prism.

**Figure S6.** Androstenedione concentration in NCI-H295R culture media upon exposure to: a) 10, 5, 1 and 0.25 mg/L of PSNP 50 nm, PSNP 150 nm, PSNP 350 nm; b) 10, 5, 1 and 0.25 mg/L PENP 50 nm, PENP 350 nm; c) 10, 5, 1 and 0.25 mg/L PPNP 50 nm, PPNP 180 nm and d) 10, 5, 1, 0.25 mg/L PET, 10 and 1 mg/L MIX50, 10, 1 and 0,5 mg/L MIX150, 10 and 1 mg/L MIX350. Results are expressed as a mean value of triplicates from two individual experiments. Statistically significant changes (* p < 0.05) relative to the negative control were calculated with one-way ANOVA and Dunnett’s multiple comparisons test in GraphPad Prism.

**Figure S7.** 17-OH-Progesterone concentration in NCI-H295R culture media upon exposure to: a) 10, 5, 1 and 0.25 mg/L of PSNP 50 nm, PSNP 150 nm, PSNP 350 nm; b) 10, 5, 1 and 0.25 mg/L PENP 50 nm, PENP 350 nm; c) 10, 5, 1 and 0.25 mg/L PPNP 50 nm, PPNP 180 nm and d) 10, 5, 1, 0.25 mg/L PET, 10 and 1 mg/L MIX50, 10, 1 and 0,5 mg/L MIX150, 10 and 1 mg/L MIX350. Results are expressed as a mean value of triplicates from two individual experiments. Statistically significant changes (* p < 0.05) relative to the negative control were calculated with one-way ANOVA and Dunnett’s multiple comparisons test in GraphPad Prism.

**Figure S8.** Progesterone concentration in NCI-H295R culture media upon exposure to: a) 10, 5, 1 and 0.25 mg/L of PSNP 50 nm, PSNP 150 nm, PSNP 350 nm; b) 10, 5, 1 and 0.25 mg/L PENP 50 nm, PENP 350 nm; c) 10, 5, 1 and 0.25 mg/L PPNP 50 nm, PPNP 180 nm and d) 10, 5, 1, 0.25 mg/L PET, 10 and 1 mg/L MIX50, 10, 1 and 0,5 mg/L MIX150, 10 and 1 mg/L MIX350. Results are expressed as a mean value of triplicates from two individual experiments. Statistically significant changes (* p < 0.05) relative to the negative control were calculated with one-way ANOVA and Dunnett’s multiple comparisons test in GraphPad Prism.

**Figure S9.** Dehydroepiandrosterone concentration in NCI-H295R culture media upon exposure to: a) 10, 5, 1 and 0.25 mg/L of PSNP 50 nm, PSNP 150 nm, PSNP 350 nm; b) 10, 5, 1 and 0.25 mg/L PENP 50 nm, PENP 350 nm; c) 10, 5, 1 and 0.25 mg/L PPNP 50 nm, PPNP 180 nm and d) 10, 5, 1, 0.25 mg/L PET, 10 and 1 mg/L MIX50, 10, 1 and 0,5 mg/L MIX150, 10 and 1 mg/L MIX350. Results are expressed as a mean value of triplicates from two individual experiments. Statistically significant changes (* p < 0.05) relative to the negative control were calculated with one-way ANOVA and Dunnett’s multiple comparisons test in GraphPad Prism.

**Figure S10.** Estradiol concentration in NCI-H295R culture media upon exposure to: a) 10, 5, 1 and 0.25 mg/L of PSNP 50 nm, PSNP 150 nm, PSNP 350 nm; b) 10, 5, 1 and 0.25 mg/L PENP 50 nm, PENP 350 nm; c) 10, 5, 1 and 0.25 mg/L PPNP 50 nm, PPNP 180 nm and d) 10, 5, 1, 0.25 mg/L PET, 10 and 1 mg/L MIX50, 10, 1 and 0,5 mg/L MIX150, 10 and 1 mg/L MIX350. Results are expressed as a mean value of triplicates from two individual experiments. Statistically significant changes (* p < 0.05) relative to the negative control were calculated with one-way ANOVA and Dunnett’s multiple comparisons test in GraphPad Prism.
